# Inhalation of *Origanum majorana* L. essential oil while working reduces perceived stress and anxiety levels of nurses in a COVID-19 intensive care unit: a randomized controlled trial

**DOI:** 10.3389/fpsyt.2023.1287282

**Published:** 2023-11-17

**Authors:** Sang Wook Lee, You Kyoung Shin, Jeong-Min Lee, Geun Hee Seol

**Affiliations:** ^1^Department of Basic Nursing Science, College of Nursing, Korea University, Seoul, Republic of Korea; ^2^KT&G Central Research Institute, Daejeon, Republic of Korea; ^3^BK21 FOUR Program of Transdisciplinary Major in Learning Health Systems, Graduate School, Korea University, Seoul, Republic of Korea

**Keywords:** marjoram, nurse, COVID-19, stress, anxiety, aromatherapy

## Abstract

**Background:**

Nurses caring for patients with coronavirus disease 2019 (COVID-19) experience higher psychosocial distress than other healthcare workers, and this can adversely affect the quality of patient care. There is evidence that inhalation of essential oil from marjoram (*Origanum majorana* L.) has calming effects, suggesting this intervention may help to reduce the stress and anxiety of nurses working in a COVID-19 intensive care unit (ICU). This study aimed to investigate the effect of inhalation of marjoram essential oil at work on the stress and anxiety levels of nurses in a COVID-19 ICU.

**Methods:**

Nurses (*n* = 57) working in a single COVID-19 ICU were randomly assigned to inhale 3% marjoram essential oil (marjoram group, *n* = 29) or almond oil (control group, *n* = 28) for 2 h while at work. Mean arterial pressure (MAP), heart rate, state anxiety score, and score on a visual analog scale for anxiety (VAS-anxiety) and stress (VAS-stress) were measured before and after the intervention.

**Results:**

The two groups had similar baseline variables. MAP did not have within-group or between-group differences. Heart rate increased significantly in the marjoram group after the intervention (*p* = 0.031), but it remained within the normal range and the increase was not clinically meaningful. There was no significant between-group difference in the state-anxiety or VAS-anxiety score after the intervention, but the marjoram group had a significantly lower state-anxiety (*p* = 0.001) and VAS-anxiety (*p* = 0.037) score at posttest vs. pretest. The VAS-stress score was significantly lower in the marjoram group at the posttest vs. the pretest (*p* = 0.026).

**Conclusion:**

Nurses caring for patients in a COVID-19 ICU experience significant stress, and strategies are needed to address this important issue. Inhalation of 3% marjoram essential oil while caring for patients in a COVID-19 ICU may be a simple and effective intervention that reduces perceived stress and anxiety in nurses.

**Clinical Trial Registration**: https://cris.nih.go.kr/, KCT0007543.

## 1 Introduction

Occupational stress in challenging psychological and physical working environments might decrease attention, concentration, and professional efficacy in healthcare workers (1). Recent studies performed in different regions have focused on mental health problems among healthcare workers during the coronavirus disease 2019 (COVID-19) pandemic. There is evidence that many factors, such as physical fatigue, emotional strain, increased workload, and concerns about disease transmission to family members, led to psychological distress in healthcare workers (2). Front-line and second-line physicians engaged in COVID-19-related clinical practice showed significantly increased presenteeism (attendance at work despite ill health) compared to those who did not, which can affect the quality of patient care (3). Notably, 29% of nurses and midwives reported suffering from moderate-to-severe anxiety, and they also had significantly higher anxiety scores than doctors. COVID-19 thus adversely affected the psychological wellbeing of healthcare workers, particularly nurses who were on the front-line of caring for patients with COVID-19 (4).

A recent study of nurses during the COVID-19 pandemic reported the pooled prevalence of stress was 43% and the pooled prevalence of anxiety was 37% (5). One study reported that pandemic-related stress was positively associated with anxiety and was a significant predictor of anxiety (6). Another study reported that relative to staff in the general ward, staff in the intensive care unit (ICU) had significantly worse quality of sleep and higher scores in three subscales of The Fear of COVID-19 Scale (7). Importantly, the psychological distress experienced by ICU nurses who were caring for critically ill patients with COVID-19 also reduced the quality of patient care (5) and increased their intention to resign from work (8). There is also evidence of suicides among nurses working in COVID-19 wards (9). Therefore, interventions are urgently required to reduce the stress and anxiety of nurses working in COVID-19 ICUs.

Several studies examined the ability of different interventions to alleviate psychological distress in nurses during the COVID-19 pandemic. For example, one study reported that the application of remote Reiki for 20 min a day for four consecutive days decreased fatigue and increased an Optimistic Approach subscale score in nurses working in a COVID-19 clinic (10). A 7-week online mindfulness-based stress reduction program increased the quality of sleep in nurses working in COVID-19 wards (11). A 20-min emotional freedom technique effectively reduced state-anxiety and stress levels in nurses caring for patients with COVID-19 (12). However, these interventions were implemented over a relatively long time (10, 11) and require several highly specific procedures (12). Therefore, there is a need for an intervention that is easier and more convenient for nurses to use in clinical settings.

Aromatherapy is a complementary therapy that uses concentrated essential oils extracted from flowers, roots, berries, and other plant parts and administers these oils by inhalation, massage, or foot bath (13). Inhalation of essential oil activates the olfactory bulb and can stimulate the hypothalamus and limbic regions, thereby providing beneficial effects on the mind and body (13). Several advantages of essential oil therapy are that the effects are nearly immediate, it is non-invasive, and it does not cause adverse effects when properly used (14).

Marjoram (*Origanum majorana* L.) is a perennial herb native to the Mediterranean region that has been widely used in traditional (non-Western) medicine to treat gastrointestinal, respiratory, and neurological diseases (15). There is some evidence that marjoram has calming effects; in that, it helps to alleviate negative emotional states such as anxiety (16). A study in mice reported that a marjoram extract effectively reduced the anxiety caused by sleep deprivation (17). Other studies found that the time spent in, and the number of entries into, the open arms in the Elevated Plus Maze test were higher in mice treated with marjoram essential oil compared to controls (18) and that mice exposed to marjoram extract spent more time in the center zone in the Open Field test compared to controls (19). In addition, inhalation of marjoram essential oil had sleep-inducing effects and reduced alpha and beta waves in humans with good sleep quality and increased theta waves in humans with poor sleep quality (20).

Thus, we hypothesized that inhalation of marjoram essential oil by nurses in a COVID-19 ICU may reduce their stress and anxiety due to its calming effects. Here, we provide the first published report examining the ability of 3% marjoram essential oil inhalation to modulate the stress and anxiety levels of nurses while at work in a COVID-19 ICU.

## 2 Materials and methods

### 2.1 Study design

This study was a double-blind randomized pretest–posttest-controlled trial. All procedures were approved by the Institutional Review Board of Korea University Guro Hospital in Seoul (2022GR0165) and were in accordance with the Declaration of Helsinki. This study was retrospectively registered in the Clinical Research Information Service (CRIS) of Korea (registration number: KCT0007543). Participants were blinded to the types and effects of the essential oil being tested, and all interventions and evaluations were conducted by the researcher blinded to patient allocations.

### 2.2 Setting and participants

Sixty eligible nurses who worked in a COVID-19 ICU at a university hospital in Korea from April 2022 to May 2022 were initially recruited. All participants were informed of the objectives and procedures of the study and provided written informed consent prior to participation. Participants were included if they (*i*) understood the objectives of the study and voluntarily agreed to participate, (*ii*) worked as nurses in the COVID-19 ICU, (*iii*) were not receiving treatment for a physical illness and were not taking an antihypertensive or anti-diabetic drug, and, and (*iv*) had no allergic reaction to marjoram essential oil. Participants were excluded if they had impaired olfactory function, were pregnant, or were breastfeeding.

**Figure 1:**
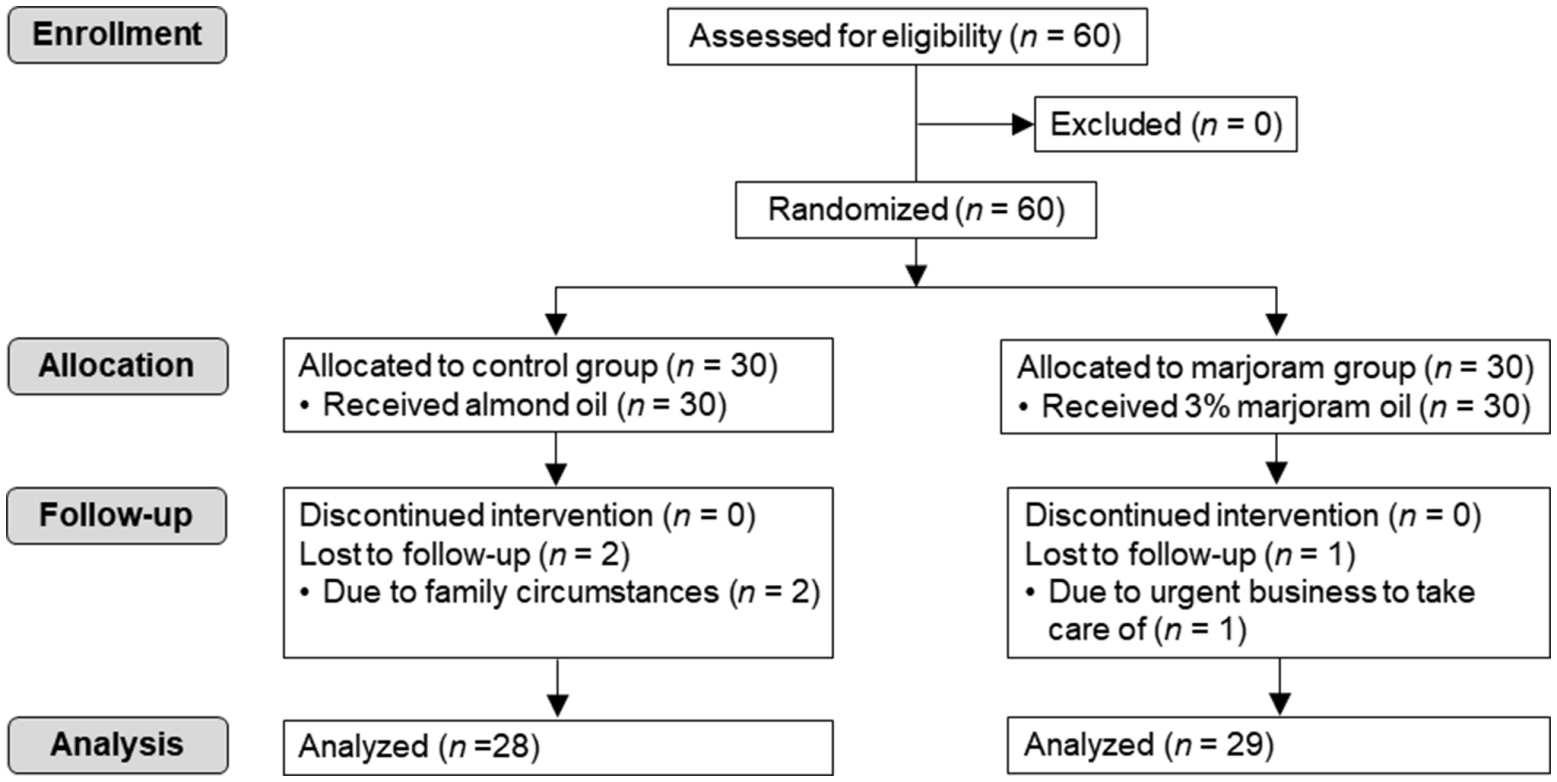
CONSORT flow diagram of this randomized pretest-posttest controlled trial.

The sample size was calculated using the G-Power program (version 3.1) to compare means in two independent groups (21). Based on a statistical power of 0.80, an effect size of 0.80, and a significance level of 0.05, the required number of participants was 26 for each group (marjoram and control). The effect size of 0.80 was calculated based on the mean and the standard deviation of a previous study that examined the effect of essential oil inhalation on stress levels in emergency nurses (22). Considering a dropout rate, 60 nurses were assigned to the control group or the marjoram group (1:1 ratio) by a simple random assignment method using Random Allocation Software (version 2.0). Independent researchers conducted the generation of the random allocation sequence and recruitment of participants to conceal the allocation sequence. Two participants in the control group and one in the marjoram group were lost to follow-up due to household circumstances or the need to attend to urgent business. Thus, 28 participants in the control group and 29 participants in the marjoram group were included in the final analysis (Figure 1).

### 2.3 Gas chromatography–mass spectrometry

Gas chromatography–mass spectroscopy (GC–MS) was used to analyze the composition of the marjoram essential oil. The utilized GC (Hewlett-Packard 5890, Palo Alto, CA, United States) had a capillary column (CP-Sil-5 CB, Chrompack, Middelburg, Netherlands) and was coupled with an analytical VG/70-250S MS instrument. Helium was used as the carrier gas, and the flow-through rate was 1.0 mL/min. Volatile compounds were identified by their retention index and retention time and were confirmed using reference samples.

### 2.4 Measurements

#### 2.4.1 Visual analog scale for stress and anxiety

Stress and anxiety levels were evaluated using separate VASs. Each VAS consisted of a straight line that was 10 cm in length, with the leftmost end corresponding to “absence of stress or anxiety” (0 points) and the rightmost end corresponding to “extreme stress or anxiety” (10 points). Each participant was asked to rate their stress level on one VAS and their anxiety level on the other by selecting a point between the two extremes. A higher score indicated greater stress or anxiety. In this study, the primary endpoint was the VAS-stress score, and the secondary endpoint was the VAS-anxiety and state-anxiety scores.

#### 2.4.2 State-anxiety score

State-anxiety scores were obtained using the Korean version of the State–Trait Anxiety Inventory (STAI) (23). The STAI consists of 20 items that assess the anxiety level of a participant at a single time point. Each item has a score ranging from 0 (“not at all”) to 4 (“very much”). The total score ranges from 0 to 80, and a higher score indicates a greater level of state-anxiety (24). Cronbach’s α for the STAI in a previous study was 0.890 (23), and in the present study was 0.925.

#### 2.4.3 Mean arterial pressure and heart rate

Psychological stress affects autonomic activity including blood pressure and heart rate (25); therefore, this study assessed blood pressure and heart rate as indices of physiological response (26). Blood pressure and heart rate were measured using an electronic sphygmomanometer (HEM-7121, Omron, Kyoto, Japan) on the right brachial artery after the participant had rested for 10 min and while seated. Measurements were conducted before and after the inhalation of the essential oil. Blood pressure status was assessed using mean arterial pressure (MAP), which was calculated as MAP = diastolic blood pressure + (systolic blood pressure − diastolic blood pressure)/3.

Mean arterial pressure provides a comprehensive measure of blood pressure status because it is influenced by systolic and diastolic blood pressure as well as pulse pressure (27).

### 2.5 Intervention

A preliminary investigation was conducted to determine the optimal concentration of marjoram essential oil. Six healthy adults who consented to participate in the preliminary testing were asked to inhale a fragrance consisting of 1, 3, 5, or 10% (v/v) marjoram essential oil (batch no. 103384; Aromarant Co., Rottingen, Germany) diluted in almond oil (Aromarant Co.). In each case, a 0.2 mL dose of marjoram essential oil was added to a 0.5 cm × 1 cm gauze pad, which was positioned 10 cm away from the nose tip. The results of the preliminary testing indicated that the optimal concentration was 3% (v/v) because the aroma remained detectable at the end of the exposure period while being non-irritative.

Prior to the intervention, all participants were evaluated for eligibility using the inclusion and exclusion criteria described above. Eligible participants were asked to complete a questionnaire that asked about their general characteristics. All measurements were performed identically before and after the intervention.

Inhalation of the essential oil began when the participant, who was wearing personal protective equipment, entered the isolation room. This personal protective equipment remained in place during the intervention. In the marjoram group, 0.2 mL of 3% (v/v) marjoram essential oil diluted in almond oil was added to a 0.5 cm × 1 cm gauze pad that was attached to the personal protective equipment at 10 cm from the nose tip. Almond oil is typically used as the carrier in aromatherapy preparations (21; 22). In the control group, 0.2 mL of almond oil alone was applied to the gauze pad. Nurses in the COVID-19 ICU worked alternately every 2 h because they were not permitted to work more than 2 h consecutively while wearing personal protective equipment (28). Thus, each participant was allowed to inhale the marjoram or control fragrance via natural breathing during a single 2-h period while in the isolation room. All interventions and evaluations were conducted by the same researcher blinded to patient allocations, from 7 AM to 9 AM, to ensure the consistency of the intervention and measurements.

### 2.6 Statistical analysis

Statistical analyses were performed using SPSS version 22.0 (SPSS Inc., Chicago, IL, United States). General characteristics were analyzed using Fisher’s exact test for categorical variables and the Student’s *t*-test or the Mann–Whitney test for continuous variables, based on the results of a normality test. Within-group differences of dependent variables before and after the intervention were analyzed using a paired *t*-test. Between-group differences were analyzed using Student’s *t*-test. A *p* value below 0.05 was considered statistically significant.

## 3 Results

### 3.1 Chemical composition of marjoram essential oil

The most abundant compound of marjoram essential oil was terpinene-4-ol (20.78%), followed by cis-4-thujanol (16.91%) and γ-terpinene (9.27%; Table 1).

**Table 1:** Gas chromatography–mass spectroscopy analysis of the commercial marjoram essential oil.

Peak no.	Compounds	RT (min)	Area	Area (%)
1	α-Pinene	5.264	29,245,844	0.77
2	β-Thujene	5.461	28,205,758	0.75
3	Sabinene	8.425	252,533,788	6.67
4	β-Myrcene	10.182	52,453,398	1.39
5	α-Terpinene	10.627	214,054,901	5.66
6	p-Cymene	11.332	79,912,073	2.11
7	β-Phellandrene	11.664	63,247,131	1.67
8	γ-Terpinene	13.365	350,910,642	9.27
9	cis-Sabinene hydrate	14.416	263,147,257	6.95
10	α-Terpinolene	14.896	102,627,346	2.71
11	trans-4-Thujanol	22.686	205,966,360	5.44
12	cis-4-Thujanol	26.172	639,736,441	16.91
13	Linalool	26.271	59,600,945	1.58
14	Linalyl acetate	26.609	181,858,109	4.81
15	trans-Caryophyllene	27.739	116,573,284	3.08
16	Terpinene-4-ol	28.296	786,441,804	20.78
17	cis-p-Menth-2-en-1-ol	29.107	47,030,232	1.24
18	α-Terpineol	31.754	183,348,733	4.85
19	Bicyclogermacrene	32.791	40,297,820	1.06
20	trans-Piperitol	33.468	28,391,311	0.75
21	trans-Ascaridol glycol	45.097	29,913,149	0.79
22	1,3-Dioxolane, 2,2-dimethyl-4,5-bis(1-methylethenyl)-	47.553	28,404,580	0.75
	Total	-	3,783,900,906	99.99

RT, Retention time.

### 3.2 General characteristics of participants and verification of group homogeneity

Among the study participants, the mean age was 26.81 years, 91% were women, the mean total ICU work experience was 34.37 months, and the mean COVID-19 ICU work experience was 11.04 months. On the standard visual analog scale (VAS), a score of 3 or less indicates low symptoms, whereas a score of 4–6 indicates medium symptoms. High symptoms are defined as a VAS score of 7 or more (29). Therefore, the participants enrolled in this study had medium anxiety and stress levels at baseline. At baseline, the marjoram and control groups had no significant difference in sex, age, work experience, MAP, heart rate, state-anxiety score, VAS-anxiety score, or VAS-stress score (Table 2).

**Table 2:** Baseline characteristics of the marjoram and control groups.

Variables	Control (*n* = 28)	Marjoram (*n* = 29)	*t* or χ^2^	*p* value
Sex				
Male	3 (10.71)	2 (6.90)	0.259	0.670^a^
Female	25 (89.29)	27 (93.10)	-	
Age (year)	26.39 ± 3.19	27.21 ± 3.88	−0.562	0.574^b^
Work experience (month)				
Total	30.25 ± 28.18	38.34 ± 37.44	−0.711	0.477^b^
COVID-19 ICU	10.18 ± 8.45	11.86 ± 8.29	−0.809	0.418^b^
MAP (mmHg)	90.99 ± 11.73	90.03 ± 9.9	0.332	0.741
Heart rate (bpm)	75.96 ± 9.78	80.59 ± 11.83	1.604	0.114
State-anxiety (score)	37.61 ± 11.15	37.59 ± 9.54	0.008	0.994
VAS-anxiety (score)	5.54 ± 1.79	5.75 ± 1.62	0.455	0.651
VAS-stress (score)	6.64 ± 1.46	6.96 ± 1.51	−0.807	0.420^b^

Data are presented as mean ± SD or *n* (%). Analyzed using Student’s *t*-test. ^a^Fisher’s exact test; ^b^Mann–Whitney test. COVID-19, Coronavirus Disease 2019; ICU, Intensive care unit; MAP, Mean arterial pressure; SD, Standard deviation; VAS, Visual analog scale.

### 3.3 Effect of the intervention on MAP and heart rate

After the intervention, there was no significant within-group or between-group difference in MAP. The marjoram group had a significantly greater heart rate at the posttest compared to the pretest (84.03 ± 12.59 vs. 80.59 ± 11.83 beat/min; *t* = −2.273, *p* = 0.031), but there was no significant between-group difference in heart rate (Table 3).

**Table 3:** Effects of the marjoram and control interventions on mean arterial pressure and heart rate.

Variables	Before Mean ± SD	After Mean ± SD	*t*	*p* value	Difference Mean ± SD	*t*	*p* value
MAP (mmHg)							
Control	90.99 ± 11.73	88.87 ± 10.28	1.738	0.094	−2.12 ± 6.45	−0.653	0.517^a^
Marjoram	90.03 ± 9.92	89.20 ± 10.06	0.549	0.587	−0.84 ± 8.22		
Heart rate (bpm)							
Control	75.96 ± 9.78	79.86 ± 13.41	−1.811	0.081	3.89 ± 11.38	−0.170	0.866^a^
Marjoram	80.59 ± 11.83	84.03 ± 12.59	−2.273	0.031	3.45 ± 8.17		

Analyzed using paired *t*-test. ^a^Student’s *t*-test. MAP, Mean arterial pressure; SD, Standard deviation.

### 3.4 Effect of the intervention on stress and anxiety levels

**Figure 2:**
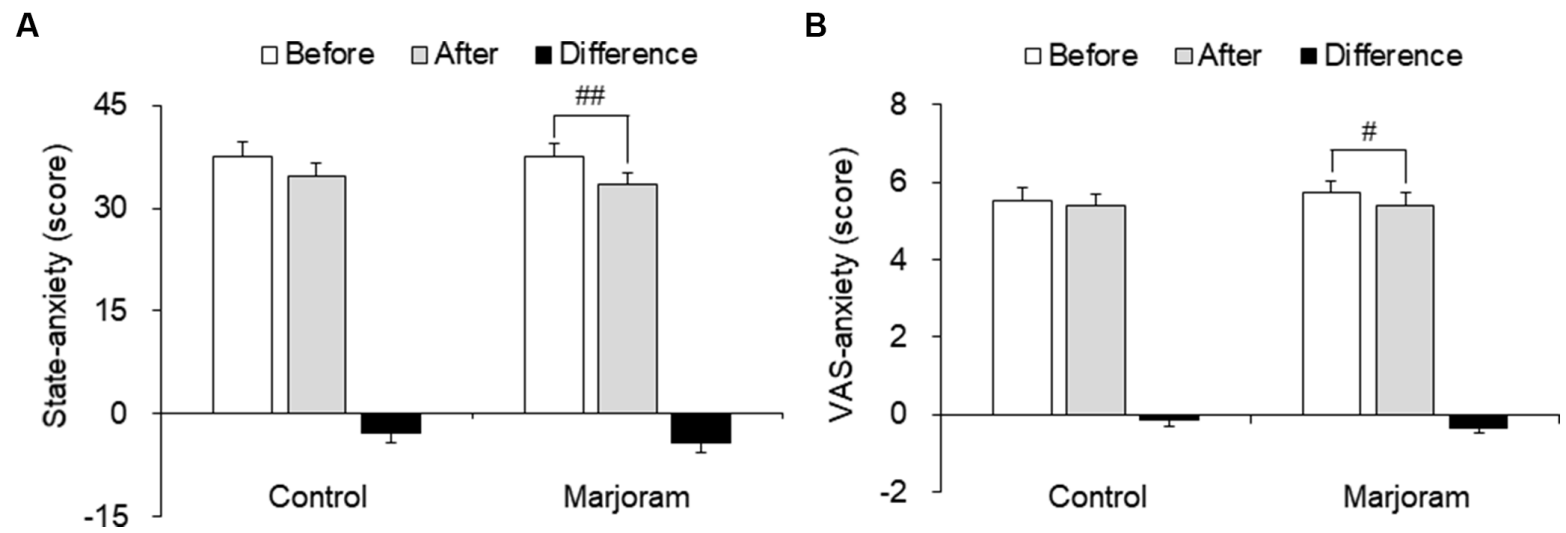
Effect of the marjoram and control interventions on the state-anxiety score **(A)** and VAS-anxiety score **(B)** Data are presented as mean ± SEM. ^#^*p* < 0.05, ^##^*p* < 0.01 vs. pretest level (within-group comparisons). VAS, visual analogue scale.

After the intervention, the control and marjoram groups did not differ significantly in total STAI score, but the marjoram group had significantly less anxiety on the two STAI sub-items, “I am worried” and “I feel pleasant.” The marjoram group also had a significantly lower total STAI score after the intervention (37.59 ± 9.54 vs. 33.38 ± 9.62; *t* = −3.148, *p* = 0.001; Figure 2A).

After the intervention, there was no significant between-group difference in the VAS-anxiety score. However, the marjoram group had a significantly lower VAS-anxiety score at posttest compared to pretest (5.75 ± 1.62 vs. 5.41 ± 0.31; *t* = 2.189, *p* = 0.037; Figure 2B).

**Figure 3:**
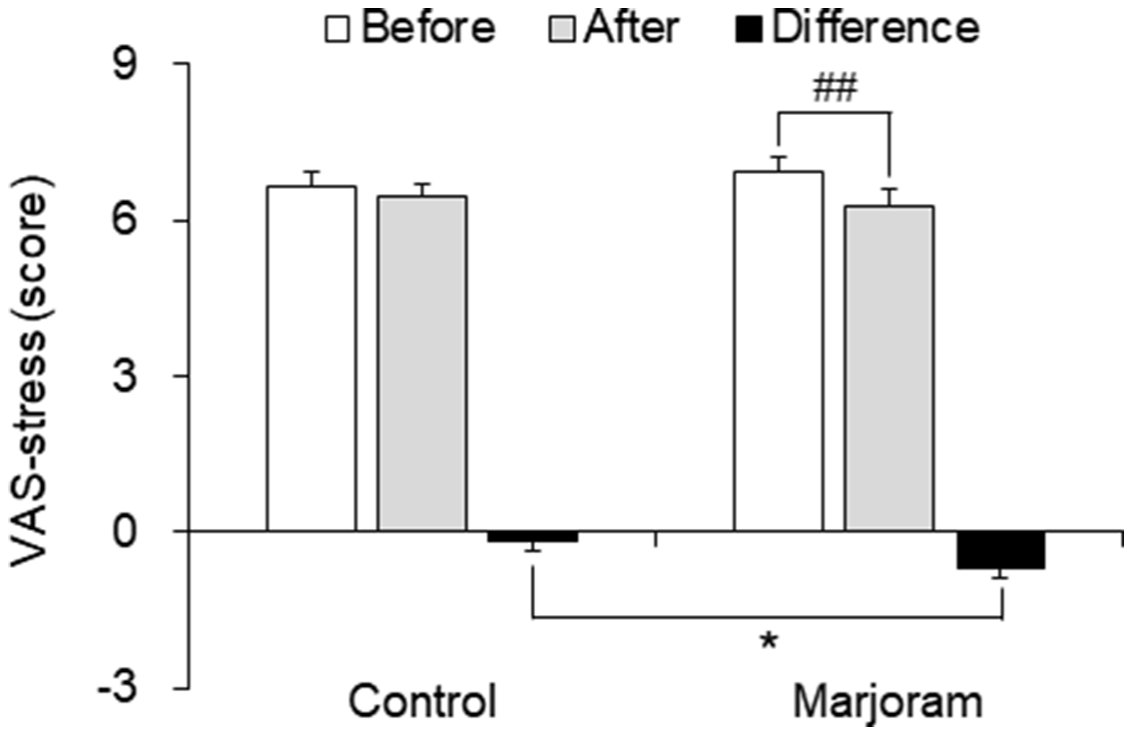
Effects of the marjoram and control interventions on VAS-stress score. Data are presented as mean ± SEM. ^##^p < 0.01 vs. pretest level (within-group comparisons); *p < 0.05 vs. control group (between-group comparisons). VAS, visual analogue scale.

The VAS-stress score declined between pretest and posttest only in the marjoram group, and this decline was significantly greater in the marjoram group compared to the control group (−0.67 ± 0.20 vs. −0.16 ± 0.18; *t* = −2.227, *p* = 0.026; Figure 3).

## 4 Discussion

To the best of our knowledge, this is the first study to examine the ability of 3% marjoram essential oil inhalation at work to modulate the stress and anxiety of nurses in a COVID-19 ICU. This safe and non-invasive aromatherapy intervention can be easily used by nurses while at work (13). We assessed its efficacy relative to a control intervention (almond oil) by measuring major clinical indicators of stress and anxiety before and after the intervention.

Mean arterial pressure and heart rate are important physiological variables, and elevated levels may be indicative of anxiety and stress. Our measurements of MAP showed no significant within- or between-group difference. The marjoram group had a significantly greater heart rate after the intervention, but the heart rate remained within the normal range and this increase was not clinically meaningful (30).

Our measurements of state- and VAS-anxiety scores revealed significant decreases in the marjoram group. A previous mouse study using the elevated plus maze test to assess anxiety found that mice treated with marjoram essential oil spent more time in the open arms and less time in the closed arms and that the anti-anxiolytic effects of marjoram essential oil were similar to those of alprazolam (18). A mouse study that used an open-field test to assess anxiety reported similar results; in that, mice exposed to a marjoram extract spent more time in the center zone (19). Consistent with these animal experiments, our findings suggest that inhalation of 3% marjoram essential oil appeared to reduce the anxiety levels of nurses working in a COVID-19 ICU.

After the intervention, the VAS-stress score was significantly lower in the marjoram group than in the control group. The VAS-stress score is appropriate for the clinical evaluation of self-reported stress levels and is widely used to measure perceived stress (31). A previous study of patients who underwent laparoscopic cholecystectomy found that the mean subjective stress level was significantly decreased after inhalation of a blend of essential oil from marjoram, lavender, ylang-ylang, and neroli, and that inhalation was also associated with increased parasympathetic nerve activity (32). Therefore, inhalation of 3% marjoram essential oil while at work may be effective in reducing the perceived stress levels of nurses working in a COVID-19 ICU.

Terpinen-4-ol is the most abundant component of marjoram essential oil (33). A study on tambaqui, a species of freshwater fish, examined how transfer to an aquarium containing terpinene-4-ol for 30 min affected these fish. The results indicated that the fish had erratic swimming and partial loss of balance, suggesting a possible calming effect (34). Therefore, our finding that inhalation of 3% marjoram essential oil reduced the perceived stress and anxiety levels of nurses in a COVID-19 ICU may be related to the alteration of autonomic nerve activity and the calming effects of marjoram essential oil.

The current study had several limitations. First, the findings cannot be easily generalized to other nurses or other healthcare professionals because the investigation was performed on nurses caring for patients in a COVID-19 ICU in a single hospital. Additionally, the sample size was relatively small although it was chosen based on a sample size calculation. Therefore, further studies are required that include larger populations of nurses or other healthcare professionals in different settings. Second, nurses in the COVID-19 ICU were not permitted to work more than 2 h consecutively while wearing personal protective equipment (3); therefore, each nurse inhaled the essential oil during a single 2-h period while in the isolation room. However, within-group differences were only observed in the anxiety scores possibly due to subjects inhaling the essential oil during only one session. Future trials are needed to investigate the effects of interventions with repeated sessions. Third, outcome variables were measured once after the intervention. However, in future studies, it may be helpful to measure the outcome variables at several time points in order to check the duration of the effects of marjoram essential oil inhalation. Finally, we did not consider an objective indicator of stress because our focus was on the effect of inhalation of marjoram essential oil on the perceived stress and anxiety levels of nurses in a COVID-19 ICU. Despite these limitations, the present findings may assist in the development of strategies for using marjoram essential oil to reduce perceived stress and anxiety in nurses caring for patients with COVID-19.

## 5 Conclusion

In conclusion, this study is the first to report that inhalation of 3% marjoram essential oil was effective in lowering the perceived stress and anxiety of nurses caring for patients with COVID-19. We suggest that inhalation of 3% marjoram essential oil is an effective, simple, and safe intervention for reducing perceived stress and anxiety levels in these nurses.

## Data availability statement

The raw data supporting the conclusions of this article will be made available by the authors, without undue reservation.

## Ethics statement

All procedures were approved by the Institutional Review Board of Korea University Guro Hospital in Seoul (2022GR0165). The studies were conducted in accordance with the local legislation and institutional requirements. The participants provided their written informed consent to participate in this study.

## Author contributions

SL: Formal analysis, Investigation, Writing – original draft. YS: Formal analysis, Visualization, Writing – original draft. J-ML: Formal analysis, Investigation, Writing - original draft. GS: Conceptualization, Formal analysis, Funding acquisition, Methodology, Project administration, Supervision, Writing – review & editing.
